# Lipid Nanoparticles Containing Mixtures of Antioxidants to Improve Skin Care and Cancer Prevention

**DOI:** 10.3390/pharmaceutics13122042

**Published:** 2021-11-30

**Authors:** Catarina Gonçalves, Maria João Ramalho, Renata Silva, Vera Silva, Rita Marques-Oliveira, Ana Catarina Silva, Maria Carmo Pereira, Joana A. Loureiro

**Affiliations:** 1 LEPABE, Department of Chemical Engineering, Faculty of Engineering, University of Porto, 4200-465 Porto, Portugal; up201608468@edu.fe.up.pt (C.G.); mjramalho@fe.up.pt (M.J.R.); 2 Associate Laboratory i4HB—Institute for Health and Bioeconomy, Faculty of Pharmacy, University of Porto, 4050-313 Porto, Portugal; rsilva@ff.up.pt (R.S.); veralssilva17@gmail.com (V.S.); ritoliveira.m@gmail.com (R.M.-O.); 3 UCIBIO—Applied Molecular Biosciences Unit, REQUIMTE, Laboratory of Toxicology, Department of Biological Sciences, Faculty of Pharmacy, University of Porto, 4050-313 Porto, Portugal; 4 UCIBIO, REQUIMTE, MEDTECH, Laboratory of Pharmaceutical Technology, Department of Drug Sciences, Faculty of Pharmacy, University of Porto, 4050-313 Porto, Portugal; 5 FP-ENAS (UFP Energy, Environment and Health Research Unit), CEBIMED (Biomedical Research Centre), Faculty of Health Sciences, University Fernando Pessoa, 4249-004 Porto, Portugal

**Keywords:** oxidative stress, drug delivery systems, bioactive compounds, solid lipid nanoparticles, nanostructured lipid carriers

## Abstract

Oxidative stress, triggered by UV radiation, is one of the major causes of free radical-associated disorders, such as skin cancer. The application of natural compounds (NCs) with antioxidant effects can attenuate free radicals’ accumulation and, therefore, provide a strategy for skin care and cancer prevention. In this work, three natural compounds, naringenin, nordihydroguaiaretic acid (NDGA), and kaempferol, were encapsulated into nanostructured lipid carriers (NLCs) aiming for the development of a formulation for cutaneous application with antioxidant properties. For the experiments, different formulation parameters were evaluated to optimize the NLCs that showed a diameter around 200 nm, which is an adequate particle size for incorporation in cosmetics. Transmission electron microscopy (TEM) analysis confirmed the NLCs’ typical spherical morphology. Encapsulation efficiency (EE) and loading capacity (LC) values revealed an effective production process, with EEs over 90% and LCs near the maximum value. The developed NLCs revealed a prolonged in vitro release of the natural compounds. The NLCs were stable under storage conditions, maintaining their psychochemical characteristics for 30 days. Additionally, they did not show any physical instability in accelerated stability studies, which also suggests long-term stability. Finally, the NCs antioxidant activity was evaluated. Interestingly, the NDGA and kaempferol mixture provided an antioxidant synergic effect. The NLC formulations’ cytotoxicity was tested in vitro in immortalized human keratinocytes (HaCaT). In addition, putative antioxidant effects of the developed NLC formulations against tert-butyl hydroperoxide (t-BHP)-induced oxidative stress were studied, and the NDGA-loaded NLC was revealed to be the one with the most protective effect. Therefore, we concluded that the naringenin, NDGA, and kaempferol incorporation into NLCs constitutes a promising strategy to increase their bioavailability and delivery to the skin.

## 1. Introduction

Skin cancer, the most common cancer type worldwide, is the out-of-control growth of abnormal cells in the epidermis, triggered by unrepaired DNA damage that leads to mutations [1]. Individual risk factors for skin cancer include age, immunosuppression, and genetic diseases, though UV radiation is the most important risk factor associated with all skin cancers [2]. The damaging UV radiation effects can be classified as direct or indirect (immunosuppression, inflammatory response, and oxidative stress). Free radicals, such as reactive oxygen species (ROS), can cause indirect damage to cellular components, and growing evidence has suggested that increased ROS levels can induce malignant cell transformation [3,4,5,6,7].

Antioxidants decrease ROS accumulation and attenuate their damaging effects. UV radiation exposure can overcome the endogenous skin protection, which is why the application of endogenous antioxidants is crucial to overcome the oxidative stress caused by an imbalance between ROS and defense mechanisms [8,9,10,11]. Natural compounds (NCs) are gaining increased attention due to their pharmacological health-benefitting properties, including antioxidant activity.

In this work, three NCs—naringenin, kaempferol, and nordihydroguaiaretic acid (NDGA)—were investigated due to their bioactive properties. Naringenin is abundant in citric fruits, such as grapefruit, oranges, as well as tomatoes, etc. Regarding its anticancer activity, NDGA exhibited dose-dependent suppressive effects of several pathways and selective cytotoxicity in different cancer cell lines [12,13,14,15,16,17]. Nevertheless, naringenin is an attractive bioactive compound mostly due to its antioxidant activity [12,13,14,15,16,17]. Additionally, some studies regarding naringenin’s effect on UV-B-radiated mice revealed a high effectiveness to protect lipids and DNA from oxidative damage, as well as reduce inflammation through the inhibition of pro-inflammatory factors. NDGA can also inhibit the ROS-mediated depletion in the levels of endogenous antioxidants [14,15]. Kaempferol can be found in plant-derived foods including beans, apples, strawberries, spinach, etc. This antioxidant presents a high capacity to decrease free radicals’ production, as well as to efficiently scavenge them. This elevated antioxidant potential can be due to the presence of its hydroxyl groups, an oxo group, and a double bond between C2 and C3 [18]. Epidemiological evidence has also shown that kaempferol can reduce the risk of developing skin, liver, breast, lung, gastric, pancreatic, and ovarian cancers [18,19]. NDGA is a polyphenolic lignan extracted from a creosote bush, Larrea tridentata. This compound has the capacity to inhibit the oxidative DNA damage and lipid peroxidation (LPO) through the inhibition of lipoxygenases (LOX). Since LPO products have been implied in the regulation of tumor cell growth, this NDGA’s capacity is also linked to its anticarcinogenic properties. NDGA also prevents oxidative damage through other mechanisms, such as activating the endogenous antioxidant system and ROS scavenging [20,21,22,23,24,25]. Moreover, these compounds possess not only potent antioxidant activity, but also anticarcinogenic, anti-inflammatory, neuroprotective, antidiabetic, and anti-aging properties, which favors their application in cosmetic formulations. These allow for oxidative stress reduction and, therefore, can be used in the prevention of many diseases such as skin cancer [13,14,18,20,21,24,26,27].

Despite being present in several consumed foods, the high hydrophobicity and low bioavailability of these natural compounds raises the need for new and innovative administration strategies to take advantage of their beneficial properties. The topical administration of these compounds is one of the surging approaches, due to the skin’s large surface area; however, transdermal penetration of active molecules is difficult due to the skin’s barrier function. To have a better chance to permeate the skin, the compounds should fulfill a certain physicochemical profile: low molecular weight, low melting point, and amphiphilic characteristics [28,29]. Additionally, it is important to consider other factors, such as compounds’ degradation at the surface, binding correctly to the skin, metabolism, etc., which make topical application more difficult [28,30].

Nanotechnology poses as an attractive field that can provide advanced solutions, improving the active compounds’ penetration through the skin [31,32,33]. Lipid nanoparticles (LNs) are the most used nanoparticles for topical application, due to their lipidic composition and, therefore, compatibility with the stratum corneum (SC) through hydrophobic interactions. Additionally, the compounds to be encapsulated in this project are hydrophobic, and LNs allow their dissolution into the lipid matrix. For cosmetic applications, the LNs’ size usually ranges between 150 and 300 nm, which minimizes systemic circulation and toxicity potential [34,35].

LNs exhibit biocompatibility with the skin, biodegradability, long-term stability, and provide formulation simplicity and versatility, which makes them an attractive option to explore in the cosmetic industry [35]. These allow for the sustained and controlled release of entrapped compounds, incrementing the bioavailability and stability by protecting of the incorporated labile compounds from degradation (light oxidation, hydrolysis, etc.), and overall allow for the skin application of molecules that are usually hard to transport [36,37].

Nanostructured lipid carriers (NLCs) are LNs composed of a matrix with solid and liquid lipids. NLCs present an unordered lipid matrix that leads to a higher degree of imperfections and, therefore, allows for a higher compound incorporation. An additional advantage of the NLCs is the minimized risk of compound expulsion over time, because the mixture of a liquid lipid with a solid lipid in the NLCs’ formulation prevents lipid recrystallization [35,36].

The goal of this work was to develop a cosmetic formulation using NLCs to simultaneously transport three potent natural antioxidant compounds (naringenin, kaempferol, and NDGA) into the skin to prevent free radical accumulation and oxidative stress that can lead to skin carcinogenesis.

## 2. Materials and Methods

### 2.1. Materials

Naringenin (N, 4′,5,7-trihydroxytlavanone, ≥98% purity, molecular weight (MW) 272.25 g/mol), kaempferol (K, 3,4′,5,7-tetrahydroxyflavone, ≥97% purity, MW 286.24 g/mol), and nordihydroguaiaretic acid (NDGA, 4-[4-(3,4-dihydroxyphenyl)-2,3-dimethylbutyl]benzene-1,2-diol, ≥97% purity, MW 302.36 g/mol) were purchased from Sigma-Aldrich (Darmstadt, Germany). Precirol^®^ 5 ATO (glyceryl distearate), Gelucire^®^ 39/01, Cetyl palmitate, Suppocire DM Pellets, Compritol^®^ H105 ATO, Gelucire^®^ 50/13 (Stearoyl polyoxyl-32 glycerides), Suppocire NA15 Pellets, Gelucire^®^ 43/01 (hard fat compounds), Apifil^®^ (PEG-8 beeswax), and Labrafac^®^ WL1349 (medium-chain triglycerides) were purchased from Gattefossé (Nanterre, France). Dynasan^®^ 114 (glyceryl tristearate), Softisan^®^ 100 (hydrogenated coco-glycerides), and Softisan^®^ 154 (hydrogenated palm oil) were provided from IOI Oleo GmbH (Hamburg, Germany). Glyceryl monostearate, stearic acid, Miglyol^®^ 812 (medium-chain triglycerides), Cetyol^®^ V (decyl oleate), isopropyl myristate, and Microcare^®^ (cetyl dimeticone) were purchased from Acopharma S.A. (Terrassa, Spain). Pluronic^®^ F-127 (MW 12,600 g/mol), HEPES hemisodium salt, pH 7.4, (MW 249.30 g/mol), 2,2-diphenyl-1-picrylhydrazyl (DPPH, MW 394.32 g/mol), and uranyl acetate (≥98% purity, MW 424.15 g/mol) were also purchased from Sigma-Aldrich (Darmstadt, Germany). Dulbecco’s modified Eagle’s medium (DMEM) with 4.5 g/L glucose and GlutaMAX™, fetal bovine serum (FBS), 0.25% trypsin/1 mM EDTA, and Hanks’ balanced salt solution (HBSS) without calcium and magnesium (HBSS (−/−)) were purchased from Gibco^TM^ (Thermo Fisher Scientific, Alfagene, Portugal). Antibiotics (100 U/mL penicillin, 100 μg/mL streptomycin) were obtained from Biochrom (Berlin, Germany). Dimethyl sulfoxide (DMSO) was purchased from Merck (Darmstadt, Germany). Neutral red (NR) solution, tert-butyl hydroperoxide (t-BHP) solution, 2′,7′-Dichlorofluorescin diacetate (DCFH-DA), and Triton™ X-100 detergent solution were acquired from Sigma-Aldrich (Darmstadt, Germany). All sterile plastic material was obtained from Corning Costar (New York, NY, USA).

### 2.2. Methods

#### 2.2.1. Formulation Studies

##### Lipid Screening

When developing a LN dispersion, the choice of the most suitable lipids is fundamental, which leads to the formation of an appropriate solid nanoparticle matrix. The used methodology was previously described and adapted to this work [38]. Briefly, a 1:100 mass ratio of each natural compound (NC) as added to the solid lipids and the mixture was heated up to 80 °C (temperature above the solid lipid melting point) and stirred for 1 h. The solubility was determined visually, at 15 min intervals, evaluating the presence or absence of crystals/aggregates of the compounds in the lipid. For the liquid lipids, a 1:100 ratio of NC to oil was stirred magnetically for 1 h, checking for the presence or absence of the compounds’ crystals. To ensure the compatibility between the solid and liquid lipids, the solubility was tested, with a 50:50 mass ratio of each, which were also heated with magnetic stirring for 1 h. Finally, they were cooled down to room temperature (20 ± 1 °C) for solidification, and the miscibility was assayed visually.

##### Surfactants Compatibility Evaluation

Nanoparticles were prepared without the NCs to identify the most suitable surfactant. Different production conditions were also assessed: Ultra-Turrax T25 (0.5, 1, and 2 min) and sonication time (5, 10, 15, and 30 min). The produced placebo NLCs were studied and monitored over time according to their mean diameter, polydispersity index (PdI), and zeta potential (ZP) through dynamic light scattering (DLS) using a ZetaSizer Nano ZS (Malvern Instruments, Worcestershire, UK) [39]. Size measurements were performed at 25 °C with an angle of detection of 173° backscatter. The obtained values were determined from 3 measurements of 11 runs each. Zeta potential values were also determined at 25 °C, using the Smoluchowski mathematical model to obtain the data resulting from 20 runs of 3 measurements. The physical appearance of the solutions was also evaluated.

#### 2.2.2. Nanoparticle Preparation

The method was adapted from the ones performed by Tichota et al. (2014) [38,40]. A mixture of the solid lipid (with a weight of 700 mg) and liquid lipid (a weight of 300 mg) was heated to at least 5 °C above the solid lipid melting point, allowing for the solid lipid to melt completely. In the lipidic phase, 20 mg of each NC was also added. At the same time, the aqueous phase, containing an aqueous surfactant solution, was heated at the same temperature. Then, the lipid phase was dispersed in the aqueous one. An emulsion was obtained using a high-shear homogenizer (Ultra-Turrax T25, Janke and Kunkel IKA-Labortechnik, Staufen, Germany) at 13,500 rpm, and sonicated using a Vibra-Cell™ CV18 (Sonics and Materials, Newtown, CT, USA) at an amplitude of 80% and an ultrasonic frequency of 24 kHz. Then, samples were left to cool to room temperature with subtle stirring, allowing for lipid crystallization and NLC formation. The production of all NLCs was done in triplicate.

#### 2.2.3. Nanoparticle Characterization

NLCs were characterized according to their mean diameter, PdI, and ZP by DLS (Zetasizer Nano ZS, Malvern Instrument, UK) at 25 °C. Samples were diluted in ultrapure water (1:100). Measurements were run in triplicate with 11 runs each. Measurements occurred on the day after the production (day 1), and on days 7, 14, and 30, to assay the NLCs’ stability over time. NLCs were also characterized according to their morphology following the method previously described [41]. Firstly, 5 µL aliquots of each sample were deposited on a 400-mesh carbon-formvar copper grid (Agar Scientific, Essex, UK) and left to absorb for 5 min. The samples were stained with a 2% (*w*/*v*) aqueous uranyl acetate solution and left to air-dry. Transmission electron microscopy (TEM) was performed using a JEM 1400 electron microscope (JEOL, Tokyo, Japan), with an acceleration voltage of 80 kV.

#### 2.2.4. Determination of Encapsulation Efficiency and Loading Capacity

The NC-loaded NLCs were diluted in ultrapure water and then filtered (Amicon^®^ Ultra Centrifugal Filters Ultracell^®^—3 kDa, Merck Millipore Ltd., Tullagreen, Carrigtwohill; from Sigma-Aldrich Química, S.A), at 14,500× *g* and 25 °C for 3 min to separate the free compounds from the compound-loaded NLCs. A mixture of 3:7 (*v*/*v*) of the supernatant and pure ethanol was prepared and the amount of free compound was quantified by UV-Vis absorbance measurements (BioTek Synergy HT Microplate Reader, BioTek, Winuschi, VT, USA) at the characteristic wavelength of each compound (282 nm for NDGA, 289 nm for naringenin, and 367 nm for kaempferol). The amount of the free compound was calculated through a calibration curve for each compound solution. This determination was performed in triplicate. Encapsulation efficiency (EE) and loading capacity (LC) were calculated through Equations (1) and (2):

(1) $$\mathrm{EE}\;\left(\%\right)=\frac{\mathrm{Total}\;\mathrm{amount}\;\mathrm{of}\;\mathrm{added}\;\mathrm{NC}-\mathrm{Unentrapped}\;\mathrm{NC}}{\mathrm{Total}\;\mathrm{amount}\;\mathrm{of}\;\mathrm{added}\;\mathrm{NC}}\times 100$$

(2) $$\mathrm{LC}\;\left(\%\right)=\frac{\mathrm{Total}\;\mathrm{mass}\;\mathrm{of}\;\mathrm{encapsulated}\;\mathrm{NC}}{\mathrm{Total}\;\mathrm{mass}\;\mathrm{of}\;\mathrm{NLC}+\mathrm{Total}\;\mathrm{mass}\;\mathrm{of}\;\mathrm{encapsulated}\;\mathrm{NC}}\times 100$$

#### 2.2.5. In Vitro Release

The in vitro release profiles of the NC-loaded NLCs were assessed in simulated physiological environment. The NC-loaded NLCs were diluted in a 10 mM solution of HEPES hemisodium salt buffer to a volume of 5 mL and placed at 37 °C and continuous stirring of 200 rpm. At predetermined timepoints, aliquots of 200 µL were collected from the release medium. To separate the released compounds from the NLCs, the samples were ultra-centrifugated (Amicon^®^ Ultra Centrifugal Filters Ultracell^®^—3 kDa, Merck Millipore Ltd., Tullagreen, Carrigtwohill; from Sigma-Aldrich Química, S.A) at 14,500× *g* and 25 °C for 3 min. The amount of the released compound was quantified by UV-Vis absorbance as described in Section 2.2.4 (BioTek Synergy HT Microplate Reader, BioTek, Winuschi, VT, USA). This test was performed in triplicate. The percentage of the released natural compound (NC) at each time point was calculated from the following equation:

(3) $$\mathrm{Released}\;\mathrm{NC}\;\left(\%\right)=\frac{\mathrm{Amount}\;\mathrm{of}\;\mathrm{NC}\;\mathrm{at}\;\mathrm{time}\;t}{\mathrm{Total}\;\mathrm{amount}\;\mathrm{of}\;\mathrm{NC}}\times 100$$

#### 2.2.6. Antioxidant Assay

The performed method was conducted as described previously [42]. Briefly, stock methanolic solutions of each NC were prepared, as well as a 0.16 mM DPPH methanolic solution. Each NC sample was added to 175 µL of DPPH solution, to a final concentration of NC or a mixture of NCs of 4 µM, and a final volume of 200 µL. The tested mixtures were naringenin (N):NDGA (2 µM N + 2 µM NDGA), N:kaempferol (K) (2 µM N + 2 µM K), NDGA:K (2 µM NDGA + 2 µM K), and N:NDGA:K (1.3 µM N + 1.3 µM NDGA + 1.3 µM K). The mixture was vortexed for 1 min and left to stand at room temperature for 30 min in the dark, and subsequently, its absorbance was read at 517 nm. Each NC and mixture of NCs was tested in triplicate. The ability of each sample to scavenge DPPH was calculated through the following equation:

(4) $$\mathrm{Scavenging}\;\mathrm{effect}\;\left(\%\right)=\left(1-\frac{A_{\mathrm{sample}}-A_{\mathrm{sample}\;\mathrm{blank}}}{A_{\mathrm{control}}}\right)\times 100$$

where A_sample_ is the absorbance of the test sample (DPPH solution plus test sample), A_control_ represents the absorbance of the control (DPPH solution without sample), and A_sample blank_ is the absorbance of the sample only (sample without the DPPH solution).

#### 2.2.7. Accelerated Stability

Accelerated stability experiments allow one to estimate the changes that may occur during storage, anticipating stability problems over time [43]. For that purpose, NLCs were diluted in ultrapure water (1:100) to a final volume of 1 mL. The NLC samples were submitted to two 3000× *g* cycles of 30 min (Eppendorf AG 5804 centrifuge, Hamburg, Germany). After each cycle, samples were examined visually to observe the presence/absence of phase separation, creaming, or flocculation, which predicts stability problems. The size, PdI, and ZP of the samples were also evaluated by DLS. This experiment was performed in triplicate.

#### 2.2.8. In Vitro Cytotoxicity Assay and Evaluation of the Protection of the Formulations against Oxidative Stress

##### Culture of Immortalized Human Keratinocytes Cells

Immortalized human keratinocytes (HaCaT) cells were routinely cultured in 75 cm^2^ flasks using DMEM with 4.5 g/L glucose and GlutaMAX™, supplemented with 10% FBS, 100 U/mL penicillin, and 100 μg/mL streptomycin. The cells were maintained in a 5% CO2-95% air atmosphere, at 37 °C, and the cell culture medium was changed every 2 days. When at 80–90% of confluence, the cultures were passaged by trypsinization (0.25% trypsin/1 mM EDTA). For the studies, the cells were seeded in 96-well plates at a density of 20,000 cells/well. The cells used in all experiments were taken between the 42nd and 50th passages.

##### Neutral Red (NR) Uptake Assay to Evaluate Formulations’ Cytotoxicity

The cytotoxicity of the NLC formulations was evaluated in HaCaT cells, as previously described by Vaz et al. (2019) [40]. For the evaluation, 24 h after cell seeding, cells were exposed to the formulations (0–1000 µg/mL) prepared in a fresh cell culture medium. After 6 and 24 h of exposure to the tested NLC formulations, cytotoxicity was evaluated by neutral red (NR) uptake assay. Triton™ X-100 (1%) was used as positive control. Briefly, the cell culture medium was aspirated, and a fresh cell culture medium containing NR (50 μg/mL) was added. The cells were then incubated, at 37 °C, in a humidified 5% CO_2_–95% air atmosphere, for 90 min. After that, the cell culture medium was removed, and the NR dye retained by viable cells was extracted with lysis buffer (absolute ethyl alcohol/distilled water (1:1) with 5% acetic acid). The absorbance was measured at 540 nm in a multi-well plate reader (PowerWaveX BioTek Instruments, Winuschi, VT, USA). The percentage of NR uptake relative to that of the control cells (0% formulation) was used as the cytotoxicity measure. The cytotoxicity of the free drugs (0.81 µg/mL) was also evaluated by the NR uptake assay, as performed for the NLC formulations. Four independent experiments were performed in triplicate.

##### Evaluation of the Protection of the Formulations against Oxidative Stress: Effects on t-BHP-Induced Increase in ROS Levels

The NLC formulations’ in vitro putative antioxidant effect was assayed using HaCaT cells. The ability of the developed NLCs in protecting against a tert-butyl hydroperoxide (t-BHP)-induced increase in the intracellular levels of ROS and reactive nitrogen species (RNS) was evaluated. Briefly, a DCFH-DA probe was applied, and when it was in the cytoplasm, the probe was hydrolyzed, and 2′,7′-dichlorodihydrofluorescein (DCFH) was formed. When ROS/RNS are present in the medium, DCFH is oxidized into the highly fluorescent 2′,7′-dichlorofluorescein (DCF), which can be quantified and whose fluorescence intensity is proportional to the levels of ROS/RNS [44]. This way, HaCaT cells were seeded for 24 h in 96-well plates (20,000 cells/well) and, after that, the cells were pre-incubated with 20 µM of DCFH-DA, protected from light, at 37 °C, in a 5% CO_2_–95% air atmosphere for 1 h. Then, DCFH-DA was removed, and the cells were subjected to t-BHP (0–500 µM) in the presence or absence of the tested NLC formulations (50 µg/mL). The exposure occurred for 24 h at 37 °C, in a 5% CO_2_–95% air atmosphere. After that, the fluorescence was measured at 485 nm excitation and 530 nm emission wavelengths in a multi-well plate reader (PowerWaveX BioTek Instruments, Winuschi, VT, USA). Four independent experiments were performed in triplicate.

#### 2.2.9. Statistical Analysis

Statistical evaluation was made using the GraphPad Prism 8 for Windows (GraphPad Software, San Diego, CA, USA). The normality of the data distribution was evaluated using the KS, D’Agostino and Pearson omnibus and Shapiro–Wilk normality tests. For data with a parametric distribution, one-way ANOVA was used to perform the statistical comparisons, followed by the Dunnett’s multiple comparisons test. For data with a non-parametric distribution, the Kruskal–Wallis test was used to perform the statistical comparisons, followed by the Dunn’s multiple comparisons test. In the evaluation of the antioxidant effect of the formulations, the comparisons were performed using two-way ANOVA followed by the Tukey’s multiple comparisons test. Details of the performed statistical analysis are described in the figure legend. Differences were considered to be significant for *p*-values < 0.05.

## 3. Results and Discussions

### 3.1. Formulation Studies

The lipids’ selection was based on their proven reported compatibility with the skin, since the goal of this formulation was for topical use. Thus, the solubility of the natural compounds was examined in several solid Supplementary (Table S1 and Figure S1) and liquid (Supplementary Table S2 and Figure S1) lipids. Out of the 15 tested solid lipids, the ones that were revealed to be the most compatible with all three NCs were Gelucire^®^ 50/13 and Apifil^®^. Concerning the liquid lipid screening, out of the five tested oils, Miglyol^®^ 812 and Labrafac WL1349 were revealed to be the ones that were able to dissolve all three NCs. Testing the several possible combinations, the ones that showed potential for NLC formulation were Gelucire^®^ 50/13 with Labrafac WL1349 and Apifil with Miglyol^®^ 812 (Supplementary Table S3 and Figure S2).

Then unloaded NLC formulations were produced using these lipids, testing two different surfactants (Tween 80 and 10% Pluronic^®^ F-127) and different production conditions. Based on the NLCs’ physicochemical properties, the selected combination was Apifil^®^ with Miglyol^®^ 812 and 10% Pluronic^®^ F-127. The production conditions for these formulations were 30 s of Ultra-Turrax T25 and 5 min of ultrasonication at an amplitude of 80% and an ultrasonic frequency of 24 kHz. These formulations presented a normal milky-like appearance, and their physicochemical properties were analyzed over time to evaluate their stability (Table 1). The formulations maintained a constant and stable particle size (*p* > 0.05) after 6 months of approximately 200 nm, which is an adequate dimension for cosmetic formulations. They also presented a PdI below 0.3, which is an acceptable value for LNs, suggesting that no particle aggregations occurred. Regarding the ZP values, these remained stable over the studied time (*p* > 0.05). LNs are usually considered to be stable when they present ZP values above or below +30 mV and −30 mV, respectively [35]. This is valid for purely electric stabilization, but despite the NLCs not having a sufficient negative charge, the system was revealed to be stable, through there was steric hindrance caused by the non-ionic surfactant used (Pluronic^®^ F-127) [45]. This study proved the feasibility of the selected NLC formulations to be a drug-delivery system for the selected NCs, since they maintained stability for to up to 6 months.

### 3.2. Natural Compound-Loaded Nanoparticles’ Characterization and Stability Studies

NC-loaded NLCs’ physicochemical properties were evaluated over time. Measurements were conducted on the day after production, and 7, 14, and 30 days after, to evaluate the NLCs’ stability (Table 2). NLCs presented sizes close to 200 nm, which prevents their systemic circulation and is adequate for cosmetic incorporation [31,34]. The NLCs’ mean diameter did not significantly change during the 30-day study (*p* > 0.05), which proves their stability over time. Comparing these results with the ones obtained for the unloaded NLCs (Table 1), it can be observed that naringenin-loaded and NDGA-loaded NLCs presented a similar diameter to the unloaded NLCs (*p* > 0.05). Kaempferol-loaded NLCs exhibited smaller dimensions than unloaded NLCs, but their sizes are still suitable for cosmetic application (*p <* 0.05). For all the produced samples, PdI remained below 0.3, revealing that the loaded NLCs are monodisperse, which guarantees their size homogeneity distribution [46,47]. The ZP values remained constant throughout the stability study (*p* > 0.05). Additionally, naringenin- and NDGA-loaded samples exhibited ZP values that were significantly different from the ones of the unloaded NLCs (*p <* 0.05), suggesting that the encapsulation of these NCs can induce changes on the particle’s surface charge. This can be explained by the different distribution of the NCs into the NLCs: if the NCs are close to the NLCs’ surface, the ZP could be significantly affected in comparison to the unloaded NLCs. Besides, it has been reported that, during the NLCs’ production process, some of the NCs could be adsorbed in the particle’s surface, therefore changing its surface charge and ZP [48].

**Figure 1:**
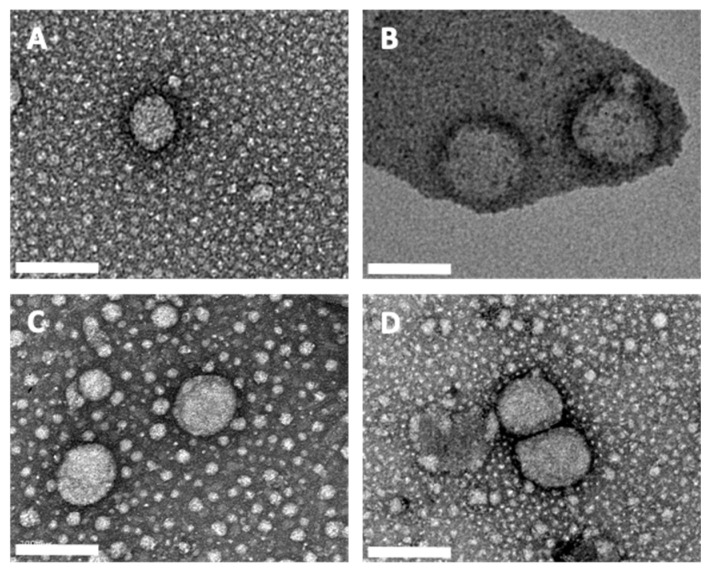
TEM images of unloaded NLCs (**A**); naringenin-loaded NLCs (**B**); NDGA-loaded NLCs (**C**), and kaempferol-loaded NLCs (**D**). Scale bars correspond to 200 nm.

Samples of unloaded and natural compound-loaded NLCs were also characterized by TEM (Figure 1). All samples revealed a typical NLC spherical morphology, as expected [49,50]. Most samples disclosed particles of approximately 200 nm, confirming the obtained DLS results. However, some discrepancies could be found, and some NLCs presented a slightly smaller size. This can be explained due to the PdI associated with every sample, and due to the impregnation technique. Additionally, other authors reported mean sizes determined by DLS to be slightly higher than by TEM due to the interference of the dispersant in the hydrodynamic diameter [51].

### 3.3. Encapsulation Efficiency and Loading Capacity

The results obtained for EE and LC values are presented in Table 3. For all samples, high EE values were obtained at over 90%, suggesting that the vast majority of the added NCs were in fact incorporated inside the LNs. All samples also revealed high LC values, near the maximum value (1.058%). These results prove that NCs’ encapsulation into LNs allows, in fact, for a suitable drug-delivery system for naringenin, NDGA, and kaempferol. These high EE and LC values are most likely due to the high NC hydrophobicity and the lipid screening that was previously performed, which had the goal to select the most compatible lipids with the three NCs. Additionally, according to the literature, the addition of the oil to the solid lipid matrix prevents its perfect crystallization, allowing for a greater drug EE and incorporation [52]. The mixture of single NC-loaded NLCs was evaluated instead of a single NLC containing the mixture of the three compounds, to avoid possible interactions between the NCs that could interfere with their activity.

### 3.4. In Vitro Release Studies

**Figure 2:**
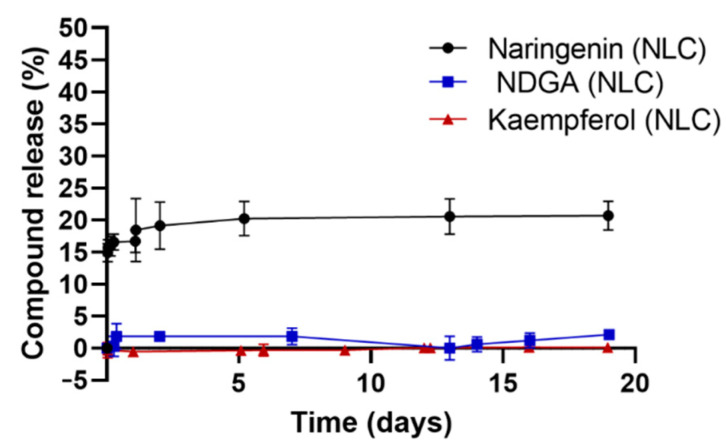
In vitro release profile of the developed NC-loaded NLCs in HEPES buffer (mean ± SD, n = 3).

The obtained in vitro release profiles for NC-loaded NLCs were evaluated for 19 days (Figure 2).

While naringenin-loaded NLCs released 21 ± 2% of their content after 19 days, NLCs containing NDGA and kaempferol revealed a very minimal release of the compounds. This prolonged release can be explained due to the NCs hydrophobicity and compatibility with the lipidic matrix due to the performed lipid screening. The high hydrophobicity and compatibility of the NCs allows for an easy incorporation of the compounds into the lipidic matrix, which provides high EE values. However, these physicochemical features of the NCs also make their release in these conditions difficult, since the used buffer is water-based. It has been described that this prolonged release occurs when the molecules are located inside the core of the nanoparticles [53,54].

Thus, the obtained results suggest that the developed NLCs are suitable for the delivery of the selected NCs for subsequent application in skin care. A slow and sustained release is advantageous for cosmetic formulations allowing for them to decrease the amount of applied formulation.

### 3.5. Antioxidant Activity Assay

The antioxidant activity of the compounds was evaluated in terms of the NCs’ ability to scavenge DPPH (Table 4). Among the three studied NCs, NDGA presented the highest antioxidant activity, and naringenin presented the lowest (*p <* 0.05). The potential antioxidant effect synergies from the combined use of more than one antioxidant were further evaluated (Table 4). The obtained results showed that the mixture containing NDGA and kaempferol was the only mixture with a synergistic effect, with significantly higher antioxidant activity values (18 ± 2) than the theoretical values (14.8) (*p <* 0.05). On the other hand, the mixture containing naringenin and kaempferol presented an antagonist effect instead of a synergistic effect, as depicted by the lower obtained antioxidant activity values (3.4 ± 0.4) than the theoretical values (4.6) (*p <* 0.05). The remaining tested combinations (NDGA plus naringenin, and NDGA plus naringenin plus kaempferol) did not present either a synergistic or an antagonist effect, since the obtained antioxidant activity values were not different for the theoretical ones (*p* > 0.05) (Table 4).

Based on the obtained results, the in vitro protective effect of the most promising mixture (NDGA plus kaempferol) was further evaluated alongside with the NCs alone in skin cells. Despite not exhibiting a synergetic effect, the mixture of the three NCs (NDGA plus naringenin plus kaempferol) was also selected to be further tested in vitro, since cell behavior could affect the antioxidant activity. Additionally, NCs possess several health-promoting properties and the combination of the three NC could still be advantageous.

### 3.6. Accelerated Stability

The results obtained for the accelerated stability assays are presented in Table 5. No significant changes were observed in the size and ZP values between the two centrifugations for all three studied nanoformulations (*p* > 0.05).

These results indicate that the centrifugations did not disrupt the NLCs nor influence their stability and homogeneity. Regarding the visual aspect of the tested samples, no physical instability (phase separation, flocculation, creaming) was observed, with the NLCs’ suspension exhibiting the same homogenous appearance as at the beginning of the experiment (Supplementary Figure S3). The obtained results can predict the long-term stability of the developed NC-loaded NLCs, suggesting that these NLCs are adequate for incorporation in cosmetic formulations.

### 3.7. In Vitro Cytotoxicity Assay and Evaluation of the Protection of the Formulations against Oxidative Stress

#### 3.7.1. Formulations Cytotoxicity

**Figure 3:**
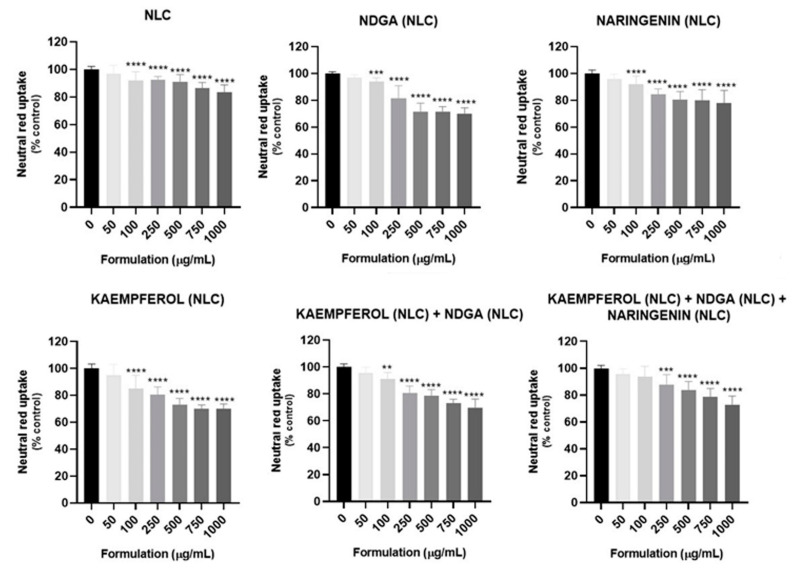
Cytotoxicity of the developed formulations evaluated in HaCaT cells by the neutral red (NR) uptake assay 24 h after exposure. Results are expressed as mean ± SD from four independent experiments performed in triplicate. Statistical comparisons were made using one-way ANOVA followed by the Dunnett’s multiple comparisons test (for data with a parametric distribution) or using the Kruskal–Wallis test followed by the Dunn’s multiple comparisons test (for data with a non-parametric distribution) ** *p* < 0.01; *** *p* < 0.001; **** *p* < 0.0001 (for each formulation vs. 0 μg/mL). In all cases, *p* values < 0.05 were considered significant.

In vitro cytotoxicity assays were performed to select the non-cytotoxic concentrations to be used in the evaluation of the antioxidant effects of the formulations against t-BHP-induced oxidative stress. The obtained results for NR uptake assay are presented in Figure 3. A concentration-dependent reduction in NR uptake was observed for all the formulations, including the unloaded NLCs. Accordingly, with the obtained data, no significant cytotoxicity was detected after 24 h of exposure to 50 µg/mL of all the tested formulations, while a significant reduction in NR uptake was observed for concentrations equal to or higher than 100 µg/mL.

**Figure 4:**
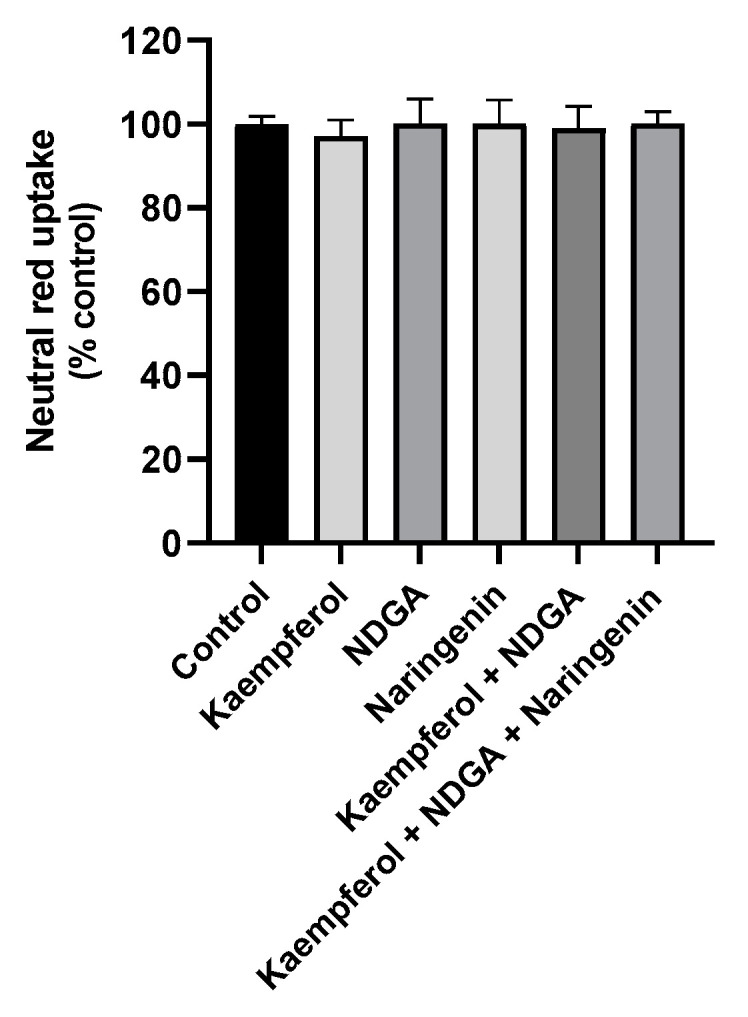
Cytotoxicity of the free natural compounds (alone or in combination) evaluated in HaCaT cells by the NR uptake assay 24 h after exposure to a 0.81 µg/mL concentration. Results are expressed as mean ± SD from four independent experiments performed in triplicate. Statistical comparisons were made using one-way ANOVA followed by the Dunnett’s multiple comparisons test. In all cases, *p* values < 0.05 were considered significant.

Based on the obtained results, the 50 µg/mL concentration was selected for the subsequent experiments, since this concentration did not present significant cytotoxicity 24 h after exposure. The cytotoxicity of the free NCs (alone or in mixtures, as in the formulations) was evaluated at the concentration of 0.81 µg/mL, the concentration of the NC present in 50 µg/mL of each formulation, to assess if the NC also did not exhibit toxicity. The obtained results are presented in Figure 4. As expected, no significant cytotoxic effects on NR uptake were detected after 24 h of contact with the tested NCs, suggesting that this concentration is, in fact, non-cytotoxic for HaCaT cells.

#### 3.7.2. Evaluation of the Protection of the Formulations against Oxidative Stress: Effects on t-BHP-Induced Increase in ROS Levels

**Figure 5:**
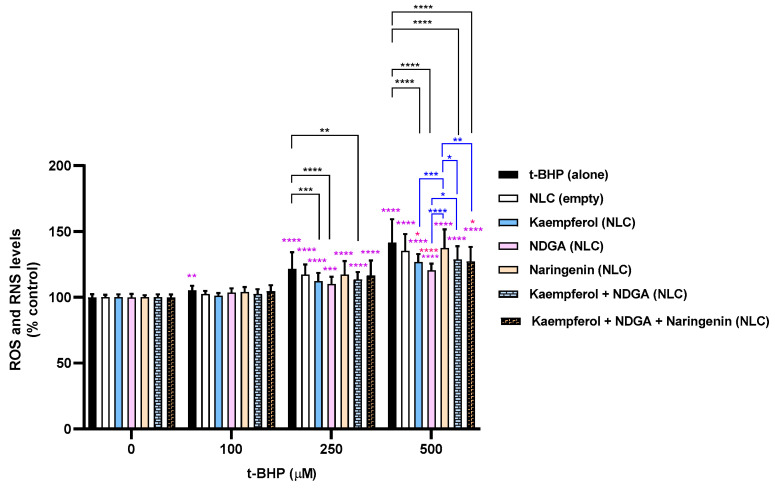
Intracellular levels of ROS/RNS 6 h after exposure to t-BHP (0–500 µM) in the presence or absence of the developed NLC formulations. Results are expressed as mean ± SD from six independent experiments performed in triplicate. Statistical comparisons were made using two-way ANOVA followed by the Tukey’s multiple comparisons test (* *p* < 0.05, ** *p* < 0.01, *** *p* < 0.001; **** *p* < 0.0001; in purple the statistical analysis for each condition versus 0 µM t-BHP is represented; in red the statistical analysis at each t-BHP concentration for the comparison between NC-loaded NLC formulations and the unloaded NLC formulation is presented; in black the statistical analysis at each t-BHP concentration for the comparison between each NLC formulation and t-BHP alone is represented; in blue the statistical analysis at each t-BHP concentration for the comparison between the different NC-loaded NLC formulations is represented). In all cases, *p*-values < 0.05 were considered significant.

**Figure 6:**
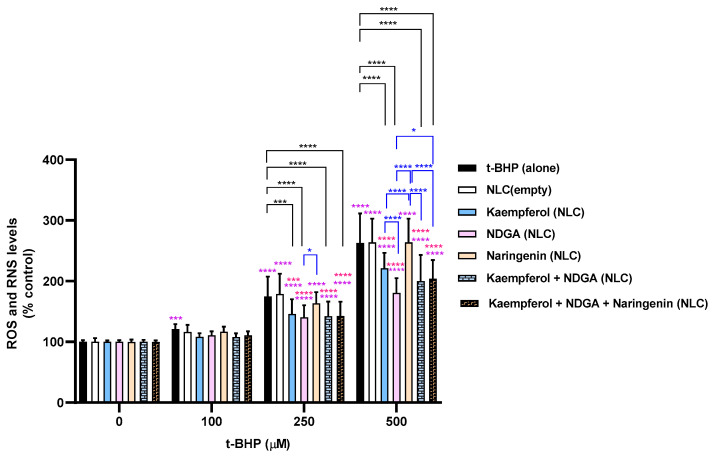
Intracellular levels of ROS/RNS 24 h after exposure to t-BHP (0–500 µM) in the presence or absence of the developed NLC formulations. Results are expressed as Mean ± SD from 6 independent experiences, performed in triplicate. Statistical comparisons were made using Two-way ANOVA followed by the Tukey’s multiple comparisons test (* *p* < 0.05, *** *p* < 0.001; **** *p* < 0.0001; in purple the statistical analysis for each condition versus 0 µM t-BHP is represented; in red the statistical analysis at each t-BHP concentration for the comparison between NC-loaded NLC formulations and unloaded NLC formulation is presented; in black the statistical analysis at each t-BHP concentration for the comparison between each NLC formulation and t-BHP alone is represented; in blue the statistical analysis at each t-BHP concentration for the comparison between the different NC-loaded NLC formulations is represented). In all cases, *p*-values < 0.05 were considered significant.

The antioxidant protective effects of the developed NLC formulations at a concentration of 50 µg/mL were assessed, and the obtained results at 6 and 24 h incubation are presented in Figure 5 and Figure 6, respectively. The mixture of NDGA-loaded plus kaempferol-loaded NLCs, and the mixture of all NLC formulations (NDGA-loaded, kaempferol-loaded, and naringenin-loaded) were also evaluated. Unloaded NLCs were used as a control.

At a 100 µM t-BHP, only a slight tendency for antioxidant activity was verified for all tested formulations and mixtures for both 6 h incubation and 24 h incubation. However, for 250 and 500 µM t-BHP, it was verified that naringenin-loaded NLCs do not have an antioxidant effect against t-BHP-induced oxidative stress, since that formulation did not significantly decrease ROS/RNS levels. These results are corroborated from the ones obtained in the antioxidant assay (Table 4), where naringenin presented lower DPPH-scavenging activity. The remaining studied formulations and mixtures exhibited antioxidant activity for 250 and 500 µM t-BHP, proved by the significant decrease in the ROS/RNS intracellular levels when compared with the control cells. These results suggest that the NLCs have the capacity to penetrate into the cells and exert their antioxidant activity. Additional studies including confocal intracellular imaging are underway to delineate penetration of the skin and intracellular localization.

Additionally, for 24 h incubation at 500 µM t-BHP, the NDGA-loaded NLC formulation was demonstrated to be the most protective formulation against t-BHP-induced oxidative stress. Indeed, in the presence of 50 µg/mL of the NDGA-loaded NLC formulation, ROS/RNS intracellular levels significantly decreased to 140.3% and 180.9% after 24 h exposure to 250 and 500 µM t-BHP, when compared to 172.3% and 256.6% of 250 and 500 µM of t-BHP alone. This is in accordance with the results obtained in the antioxidant activity assay (Table 4), where NDGA presented the strongest DPPH-scavenging activity among all the tested NCs.

Furthermore, although it was not the most efficient formulation, the mixture of the three loaded NLC formulations still proved to significantly improve the protective effects against oxidative stress. Additionally, and as expected, the empty NLC formulation was not able to decrease ROS/RNS production when compared to t-BHP alone, suggesting that the protective effects observed for the formulations are most likely a result of the antioxidant effects exerted by the NLCs loaded with the NCs. These results suggest that the developed NC-loaded NLCs are adequate for the development of novel cosmetic formulations with antioxidant activity.

## 4. Conclusions

In this work, formulations of NLCs containing three natural compounds with antioxidant activity—naringenin, NDGA, and kaempferol—were prepared. The formulations and production process were optimized to yield nanoparticles with adequate physicochemical properties for incorporation in cosmetic formulations. The developed NLCs maintained their characteristics over 30 days, revealing stability during storage. High EE and LC values were obtained for all NLCs, suggesting an efficient formulation process, with low compound loss, low production costs, and thus, the achievement of more sustainable products. The developed NLCs exhibited a prolonged in vitro release profile, which is beneficial for cosmetic formulations allowing them to decrease the amount and frequency of the applied formulation. The long-term stability of the NLC formulations, revealed by the accelerated stability assay, is also advantageous for cosmetics. The NC-loaded NLCs presented, as expected, good antioxidant activity, and the mixture containing NDGA and kaempferol revealed to be the most promising due to a synergic effect. The developed NLC formulations and free NCs were evaluated in vitro in HaCaT cells, where no significant cytotoxicity was detected after exposure to 50 µg/mL of the formulations. Finally, the antioxidant effects of the NLC formulations against induced oxidative stress were studied in vitro in the same cells. Although the mixtures of all NC-loaded NLCs showed significant antioxidant effects, the NDGA-loaded NLCs presented the highest protective effect against t-BHP-induced oxidative stress. However, despite not exhibiting the strongest protective effect, the mixture of the three loaded NLC formulations still proved to confer protection against the oxidative stress, and therefore has the potential to be used for cosmetic applications alongside with NDGA-loaded NLCs. Furthermore, the mixture of the selected molecules could benefit from the other therapeutic activities of each natural compound, such as anticarcinogenic, anti-inflammatory, and anti-aging activities.

The results of this work suggest that lipid nanoparticles, in particular NLCs, are promising carriers to deliver the combination of naringenin, NDGA, and kaempferol to the skin, improving their bioavailability. This paves the way for new cosmetic products with health-promoting properties for skin care and skin cancer prevention. This formulation can be further incorporated into a hydrogel, to facilitate cutaneous application, and tested in human volunteers. Besides, the developed formulation could have different fields of application, e.g., for neurodegenerative disorders prevention, since oxidative stress is closely related with these diseases.

## Figures and Tables

**Table 1:** The mean diameter (size), polydispersity index (PdI), and zeta potential (ZP) of the unloaded NLC formulations (mean ± SD, n = 3).

Measurement	NLC		
	Size (nm)	PdI	ZP (mV)
Day 1	222 ± 13	0.231 ± 0.005	−14.4 ± 0.9
Day 7	229 ± 10	0.220 ± 0.014	−15.2 ± 1.7
Day 14	227 ± 8	0.264 ± 0.075	−14.2 ± 0.7
Month 6	225 ± 9	0.215 ± 0.012	−15.9 ± 0.6

**Table 2:** The mean diameter (size), polydispersity index (PdI), and zeta potential (ZP) of the naringenin-, NDGC-, and kaempferol-loaded NLCs (mean ± SD, n = 3).

Measurement	Naringenin			NDGA			Kaempferol		
	Size (nm)	PdI	ZP (mV)	Size (nm)	PdI	ZP (mV)	Size (nm)	PdI	ZP (mV)
Day 1	208 ± 3	0.223 ± 0.017	−9.0 ± 0.3	213 ± 4	0.234 ± 0.007	−22.9 ± 4.1	176 ± 7	0.221 ± 0.002	−12.0 ± 2.2
Day 7	204 ± 7	0.205 ± 0.011	−10.1 ± 0.9	207 ± 9	0.224 ± 0.009	−20.8 ± 0.4	180 ± 8	0.219 ± 0.013	−8.9 ± 0.8
Day 14	204 ± 11	0.205 ± 0.004	−9.7 ± 0.3	204 ± 6	0.234 ± 0.026	−20.3 ± 0.8	180 ± 7	0.221 ± 0.007	−14.5 ± 0.8
Day 30	204 ± 6	0.205 ± 0.004	−12.0 ± 1.8	207 ± 5	0.214 ± 0.002	−19.9 ± 1.1	182 ± 6	0.209 ± 0.005	−13.1 ± 0.7

**Table 3:** Encapsulation efficiencies and loading capacity of naringenin, NDGA, and kaempferol in the NLCs (mean ± SD, n = 3).

	Naringenin	NDGA	Kaempferol
Encapsulation efficiency (%)	91.1 ± 1.7	89.8 ± 1.9	98.8 ± 0.7
Loading capacity (%)	0.97 ± 0.02	0.95 ± 0.02	1.05 ± 0.01

**Table 4:** DPPH radical-scavenging activities of natural compounds. NDGA (4 µM), naringenin (N) (4 µM), and kaempferol (K) (4 µM), as well as mixtures of NDGA and N (2 µM NDGA + 2 µM N), NDGA and K (2 µM NDGA + 2 µM K), N and K (2 µM N + 2 µM K), and NDGA and N and K (1.3 µM NDGA + 1.3 µM N + 1.3 µM K) (mean ± SD, n = 3).

Antioxidant Activity (%)	NDGA	N	K	NDGA:N	NDGA:K	N:K	NDGA:N:K
Obtained	21.8 ± 1.6	1.4 ± 1.3	7.8 ± 1.4	12.3 ± 1.2	17.8 ± 1.7	3.4 ± 0.4	10.7 ± 0.7
Theoretical	-	-	-	11.6	14.8	4.6	10.3

**Table 5:** The mean diameter (size), polydispersity index (PdI), and zeta potential (ZP) of the naringenin-, NDGA-, and kaempferol-loaded NLCs after the accelerated stability assay (mean ± SD, n = 3).

Measurement	Naringenin			NDGA			Kaempferol		
	Size (nm)	PdI	ZP (mV)	Size (nm)	PdI	ZP (mV)	Size (nm)	PdI	ZP (mV)
After 30 min centrifugation	197 ± 8	0.210 ± 0.013	−12.1 ± 0.5	208 ± 9	0.221 ± 0.006	−21.3 ± 1.2	186 ± 10	0.206 ± 0.017	−9.5 ± 0.9
After 60 min centrifugation	198 ± 7	0.193 ± 0.023	−12.8 ± 0.4	208 ± 8	0.221 ± 0.008	−22.3 ± 0.4	186 ± 4	0.209 ± 0.024	−10.3 ± 2.9

## Supplementary Materials

The following are available online at https://www.mdpi.com/article/10.3390/pharmaceutics13122042/s1, Table S1: Solid lipid Screening; Table S2: Liquid lipid screening; Table S3: Solid and liquid lipid compatibility assay; Figure S1: Examples of lipids that were able to solubilize the NC; Figure S2: Compatibility evaluation between the solid and liquid lipids; Figure S3: Images of the samples before and after the first and second centrifugation included in the accelerated stability study assays before centrifugation.

## References

[1] Foundation S.C. Skin Cancer Information. https://www.skincancer.org/skin-cancer-information/.

[2] Didona D., Paolino G., Bottoni U., Cantisani C. (2018). Non melanoma skin cancer pathogenesis overview. Biomedicines.

[3] Poljšak B., Dahmane R. (2012). Free radicals and extrinsic skin aging. Dermatol. Res. Pract..

[4] Swalwell H., Latimer J., Haywood R.M., Birch-Machin M.A. (2012). Investigating the role of melanin in UVA/UVB-and hydrogen peroxide-induced cellular and mitochondrial ROS production and mitochondrial DNA damage in human melanoma cells. Free Radic. Biol. Med..

[5] Herrling T., Jung K., Fuchs J. (2006). Measurements of UV-generated free radicals/reactive oxygen species (ROS) in skin. Spectrochim. Acta Part. A Mol. Biomol. Spectrosc..

[6] Harris I.S., DeNicola G.M. (2020). The complex interplay between antioxidants and ROS in cancer. Trends Cell Biol..

[7] Tharkar P., Varanasi R., Wong W.S.F., Jin C.T., Chrzanowski W. (2019). Nano-enhanced drug delivery and therapeutic ultrasound for cancer treatment and beyond. Front. Bioeng. Biotechnol..

[8] Poljsak B., Šuput D., Milisav I. (2013). Achieving the balance between ROS and antioxidants: When to use the synthetic antioxidants. Oxid. Med. Cell. Longev..

[9] Poljsak B., Glavan U., Dahmane R. (2011). Skin cancer, free radicals and antioxidants. Int. J. Cancer Res. Prev..

[10] Kusumawati I., Indrayanto G. (2013). Natural antioxidants in cosmetics. Studies in Natural Products Chemistry.

[11] Godic A., Poljšak B., Adamic M., Dahmane R. (2014). The role of antioxidants in skin cancer prevention and treatment. Oxid. Med. Cell. Longev..

[12] Martinez R.M., Pinho-Ribeiro F.A., Steffen V.S., Silva T.C., Caviglione C.V., Bottura C., Fonseca M.J., Vicentini F.T., Vignoli J.A., Baracat M.M. (2016). Topical formulation containing naringenin: Efficacy against ultraviolet B irradiation-induced skin inflammation and oxidative stress in mice. PLoS ONE.

[13] Joshi R., Kulkarni Y.A., Wairkar S. (2018). Pharmacokinetic, pharmacodynamic and formulations aspects of Naringenin: An update. Life Sci..

[14] Martinez R.M., Pinho-Ribeiro F.A., Steffen V.S., Caviglione C.V., Vignoli J.A., Barbosa D.C.S., Baracat M.M., Georgetti S.R., Verri W.A., Casagrande R. (2015). Naringenin inhibits UVB irradiation-induced inflammation and oxidative stress in the skin of hairless mice. J. Nat. Prod..

[15] Kumar R., Bhan Tiku A. (2020). Naringenin Suppresses Chemically Induced Skin Cancer in Two-Stage Skin Carcinogenesis Mouse Model. Nutr. Cancer.

[16] Zaidun N.H., Thent Z.C., Abd Latiff A. (2018). Combating oxidative stress disorders with citrus flavonoid: Naringenin. Life Sci..

[17] Cavia-Saiz M., Busto M.D., Pilar-Izquierdo M.C., Ortega N., Perez-Mateos M., Muñiz P. (2010). Antioxidant properties, radical scavenging activity and biomolecule protection capacity of flavonoid naringenin and its glycoside naringin: A comparative study. J. Sci. Food Agric..

[18] Calderon-Montano J.M., Burgos-Morón E., Pérez-Guerrero C., López-Lázaro M. (2011). A review on the dietary flavonoid kaempferol. Mini Rev. Med. Chem..

[19] Imran M., Salehi B., Sharifi-Rad J., Aslam Gondal T., Saeed F., Imran A., Shahbaz M., Tsouh Fokou P.V., Umair Arshad M., Khan H. (2019). Kaempferol: A key emphasis to its anticancer potential. Molecules.

[20] Manda G., Rojo A.I., Martínez-Klimova E., Pedraza-Chaverri J., Cuadrado A. (2020). Nordihydroguaiaretic Acid: From Herbal Medicine to Clinical Development for Cancer and Chronic Diseases. Front. Pharmacol..

[21] Hernández-Damián J., Andérica-Romero A.C., Pedraza-Chaverri J. (2014). Paradoxical cellular effects and biological role of the multifaceted compound nordihydroguaiaretic acid. Arch. Der Pharm..

[22] Paracatu L.C., de Faria C.M.Q.G., Zeraik M.L., Quinello C., Rennó C., Palmeira P., da Fonseca L.M., Ximenes V.F. (2015). Hydrophobicity and antioxidant activity acting together for the beneficial health properties of nordihydroguaiaretic acid. Food Funct..

[23] Galano A., Macías-Ruvalcaba N.A., Medina Campos O.N., Pedraza-Chaverri J. (2010). Mechanism of the OH radical scavenging activity of nordihydroguaiaretic acid: A combined theoretical and experimental study. J. Phys. Chem. B.

[24] Lü J.-M., Nurko J., Weakley S.M., Jiang J., Kougias P., Lin P.H., Yao Q., Chen C. (2010). Molecular mechanisms and clinical applications of nordihydroguaiaretic acid (NDGA) and its derivatives: An update. Med. Sci. Monit. Int. Med. J. Exp. Clin. Res..

[25] Floriano-Sánchez E., Villanueva C., Medina-Campos O.N., Rocha D., Sanchez-González D.J., Cardenas-Rodríguez N., Pedraza-Chaverrí J. (2006). Nordihydroguaiaretic acid is a potent in vitro scavenger of peroxynitrite, singlet oxygen, hydroxyl radical, superoxide anion and hypochlorous acid and prevents in vivo ozone-induced tyrosine nitration in lungs. Free Radic. Res..

[26] Chen A.Y., Chen Y.C. (2013). A review of the dietary flavonoid, kaempferol on human health and cancer chemoprevention. Food Chem..

[27] Yao K., Chen H., Liu K., Langfald A., Yang G., Zhang Y., Yu D.H., Kim M.O., Lee M.-H., Li H. (2014). Kaempferol targets RSK2 and MSK1 to suppress UV radiation-induced skin cancer. Cancer Prev. Res..

[28] Touitou E. (2002). Drug delivery across the skin. Expert Opin. Biol. Ther..

[29] Zsikó S., Csányi E., Kovács A., Budai-Szűcs M., Gácsi A., Berkó S. (2019). Methods to evaluate skin penetration in vitro. Sci. Pharm..

[30] Nair A., Jacob S., Al-Dhubiab B., Attimarad M., Harsha S. (2013). Basic considerations in the dermatokinetics of topical formulations. Braz. J. Pharm. Sci..

[31] Garcês A., Amaral M., Lobo J.S., Silva A. (2018). Formulations based on solid lipid nanoparticles (SLN) and nanostructured lipid carriers (NLC) for cutaneous use: A review. Eur. J. Pharm. Sci..

[32] Gupta S., Bansal R., Gupta S., Jindal N., Jindal A. (2013). Nanocarriers and nanoparticles for skin care and dermatological treatments. Indian Dermatol. Online J..

[33] Ramalho M.J., Andrade S., Loureiro J.A., do Carmo Pereira M. (2020). Nanotechnology to improve the Alzheimer’s disease therapy with natural compounds. Drug Deliv. Transl. Res..

[34] H Muller R., Shegokar R., M Keck C. (2011). 20 years of lipid nanoparticles (SLN & NLC): Present state of development & industrial applications. Curr. Drug Discov. Technol..

[35] Tamjidi F., Shahedi M., Varshosaz J., Nasirpour A. (2013). Nanostructured lipid carriers (NLC): A potential delivery system for bioactive food molecules. Innov. Food Sci. Emerg. Technol..

[36] Müller R.H., Radtke M., Wissing S.A. (2002). Solid lipid nanoparticles (SLN) and nanostructured lipid carriers (NLC) in cosmetic and dermatological preparations. Adv. Drug Deliv. Rev..

[37] Khater D., Nsairat H., Odeh F., Saleh M., Jaber A., Alshaer W., Al Bawab A., Mubarak M.S. (2021). Design, Preparation, and Characterization of Effective Dermal and Transdermal Lipid Nanoparticles: A Review. Cosmetics.

[38] Tichota D.M., Silva A.C., Lobo J.M.S., Amaral M.H. (2014). Design, characterization, and clinical evaluation of argan oil nanostructured lipid carriers to improve skin hydration. Int. J. Nanomed..

[39] Ramalho M.J., Loureiro J.A., Coelho M.A.N., Pereira M.C. (2019). Factorial design as a tool for the optimization of plga nanoparticles for the co-delivery of temozolomide and o6-benzylguanine. Pharmaceutics.

[40] Vaz S., Silva R., Amaral M., Martins E., Lobo J.S., Silva A. (2019). Evaluation of the biocompatibility and skin hydration potential of vitamin E-loaded lipid nanosystems formulations: In vitro and human in vivo studies. Colloids Surf. B Biointerfaces.

[41] Loureiro J.A., Andrade S., Duarte A., Neves A.R., Queiroz J.F., Nunes C., Sevin E., Fenart L., Gosselet F., Coelho M.A. (2017). Resveratrol and grape extract-loaded solid lipid nanoparticles for the treatment of Alzheimer’s disease. Molecules.

[42] Rahman N.A., Katayama T., Wahid M.E.A., Kasan N.A., Khatoon H., Yamada Y., Takahashi K. (2020). Taxon- and Growth Phase-Specific Antioxidant Production by Chlorophyte, Bacillariophyte, and Haptophyte Strains Isolated From Tropical Waters. Front. Bioeng. Biotechnol..

[43] Eiras F., Amaral M., Silva R., Martins E., Lobo J.S., Silva A. (2017). Characterization and biocompatibility evaluation of cutaneous formulations containing lipid nanoparticles. Int. J. Pharm..

[44] He Y.-Y., Häder D.-P. (2002). Involvement of reactive oxygen species in the UV-B damage to the cyanobacterium *Anabaena* sp.. J. Photochem. Photobiol. B Biol..

[45] Loureiro J.A., Rocha S., Pereira M.D.C. (2013). Charged surfactants induce a non-fibrillar aggregation pathway of amyloid-beta peptide. J. Pept. Sci..

[46] Subramaniam B., Siddik Z.H., Nagoor N.H. (2020). Optimization of nanostructured lipid carriers: Understanding the types, designs, and parameters in the process of formulations. J. Nanoparticle Res..

[47] Malta R., Loureiro J.B., Costa P., Sousa E., Pinto M., Saraiva L., Amaral M.H. (2021). Development of lipid nanoparticles containing the xanthone LEM2 for topical treatment of melanoma. J. Drug Deliv. Sci. Technol..

[48] Asasutjarit R., Lorenzen S.-I., Sirivichayakul S., Ruxrungtham K., Ruktanonchai U., Ritthidej G.C. (2007). Effect of solid lipid nanoparticles formulation compositions on their size, zeta potential and potential for in vitro pHIS-HIV-hugag transfection. Pharm. Res..

[49] Krambeck K., Silva V., Silva R., Fernandes C., Cagide F., Borges F., Santos D., Otero-Espinar F., Lobo J.M.S., Amaral M.H. (2021). Design and characterization of Nanostructured lipid carriers (NLC) and Nanostructured lipid carrier-based hydrogels containing Passiflora edulis seeds oil. Int. J. Pharm..

[50] Pinto C.M., Horta L.S., Soares A.P., Carvalho B.A., Ferreira E., Lages E.B., Ferreira L.A.M., Faraco A.A.G., Santiago H.C., Goulart G.A.C. (2021). Nanoencapsulated Doxorubicin Prevents Mucositis Development in Mice. Pharmaceutics.

[51] Souza T.G., Ciminelli V.S., Mohallem N.D.S. (2016). A comparison of TEM and DLS methods to characterize size distribution of ceramic nanoparticles. J. Phys. Conf. Ser..

[52] Souto E.B., Wissing S.A., Barbosa C.M., Müller R.H. (2004). Development of a controlled release formulation based on SLN and NLC for topical clotrimazole delivery. Int. J. Pharm..

[53] Borges A., de Freitas V., Mateus N., Fernandes I., Oliveira J. (2020). Solid lipid nanoparticles as carriers of natural phenolic compounds. Antioxidants.

[54] Baek J.-S., Cho C.-W. (2015). Controlled release and reversal of multidrug resistance by co-encapsulation of paclitaxel and verapamil in solid lipid nanoparticles. Int. J. Pharm..

