# Supplementary material for: Lipid Nanoparticles Containing Mixtures of Antioxidants to Improve Skin Care and Cancer Prevention
Source: Pharmaceutics. 2021 Nov 30;13(12):2042. doi: 10.3390/pharmaceutics13122042 (PMC8706926; doi:10.3390/pharmaceutics13122042)
Supplement: Supplementary file 1 [file pharmaceutics-13-02042-s001.zip › pharmaceutics-1443476-supplementary.pdf]

pharmaceutics

MDPI

Supplementary Material: Lipid Nanoparticles Containing Mixtures of Antioxidants to Improve Skin Care and Cancer Prevention

Catarina Gonçalves [1], Maria João Ramalho [1], Renata Silva [2], Vera Silva [2], Rita Marques-Oliveira [2], Ana Catarina Silva [3,4,*], Maria Carmo Pereira [1,*] and Joana A. Loureiro [1,*]

[1] LEPABE, Department of Chemical Engineering, Faculty of Engineering of the University of Porto, 4200-465Porto, Portugal; up201608468@edu.fe.up.pt (C.G.); mjramalho@fe.up.pt (M.J.R.)
[2] UCIBIO, REQUIMTE, Laboratory of Toxicology, Department of Biological Sciences, Faculty of Pharmacy, University of Porto, 4050-313 Porto, Portugal; rsilva@ff.up.pt (R.S.); veralssilva17@gmail.com (V.S.); ritoliveira.m@gmail.com (R.M.-O.)
[3] UCIBIO, REQUIMTE, MEDTECH, Laboratory of Pharmaceutical Technology, Department of Drug Sciences, Faculty of Pharmacy, University of Porto, 4050-313 Porto, Portugal
[4] FP-ENAS (UFP Energy, Environment and Health Research Unit), CEBIMED (Biomedical Research Centre), Faculty of Health Sciences, University Fernando Pessoa, 4249-004 Porto, Portugal
* Correspondence: ana.silva@ff.up.pt (A.C.S.); mcsp@fe.up.pt (M.C.P.); jasl@fe.up.pt (J.A.L.)

Table S1. Solid lipid Screening.

	Naringenin					NDGA					Kaempferol				
		Time (min)					Time (min)					Time (min)			
**Solid lipid**	**Ratio (w/w)**	**15**	**30**	**45**	**60**	**Ratio (w/w)**	**15**	**30**	**45**	**60**	**Ratio (w/w)**	**15**	**30**	**45**	**60**
**Gelucire® 50/13**	1:100	-	-	-	-	1:100	-	-	-	-	1:100	+	-	-	-
**Softisan® 100**	1:100	+	-	-	-	1:100	+	+	-	-	1:100	+	+	+	+
**Com-pritol® H105 ATO**	1:100	-	-	-	-	1:100	+	-	-	-	1:100	+	+	+	+
**Apifil®**	1:100	+	-	-	-	1:100	-	-	-	-	1:100	-	-	-	-
**Precirol® 5 ATO**	1:200	-	-	-	-	1:100	-	-	-	-	1:100	+	+	+	+
**Witepsol® E76**	1:200	+	-	-	-	1:100	+	+	+	+					
**Suppocire DM Pel-lets**	1:200	+	+	-	-	1:100	+	+	+	+					
**Suppocire NA15 pel-lets**	1:200	+	+	-	-	1:100	+	+	+	+					

**Gelucire 39/01**	1:200	+	+	-	-	1:100	+	+	+	+					
**Gelucire® 43/01**	1:200	+	+	-	-	1:100	+	+	+	+					
**Cetyl palmitate**	1:200	+	+	-	-	1:100	+	+	+	+					
**Stearic acid**	1:200	+	+	-	-	1:100	+	+	+	+					
**Dynasan® 118**	1:100	+	+	+	+										
**Glyceryl monostearate**	1:100	+	+	+	+										
**Softisan® 154**	1:200	+	+	+	+										

Table S2. Liquid lipid screening.

	**Naringenin**					**NDGA**					**Kaempferol**				
		**Time (min)**					**Time (min)**					**Time (min)**			
**Solid lipid**	**Ratio (w/w)**	**15**	**30**	**45**	**60**	**Ratio (w/w)**	**15**	**30**	**45**	**60**	**Ratio (w/w)**	**15**	**30**	**45**	**60**
**Miglyol 812 (medium-chain triglycerides)**	1:100	+	-	-	-	1:100	-	-	-	-	1:200	-	-	-	-
**Labrafac WL1349 (medium-chain triglycerides)**	1:100	+	-	-	-	1:100	-	-	-	-	1:200	-	-	-	-
**Microcare (cetyl dimeticone)**	1:100	+	+	-	-	1:100	+	+	+	+					
**Isopropyl myristate**	1:100	+	+	+	+										
**Cetyol V (decyl oleate)**	1:100	+	+	+	+										

Table S3. Solid and liquid lipid compatibility assay.

Solid lipid \ Liquid lipid	Miglyol® 812	Labrafac WL1349
**Gelucire® 50/13**	+	-
**Apifil®**	-	+

Solid lipid screening for kaempferol in a 1:100 proportion: (a) Apifil®; and (b) Precirol® 5 ATO

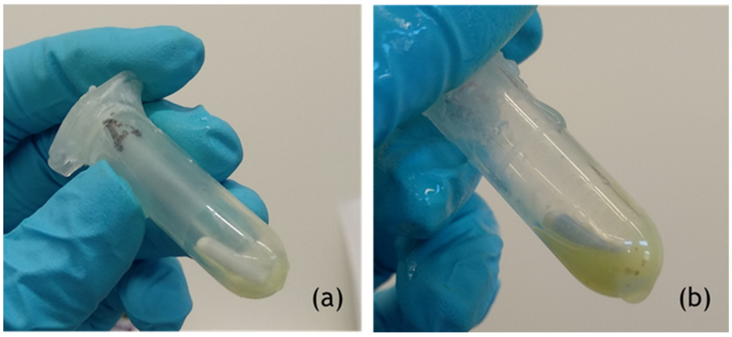

Liquid lipid screening for naringenin in a 1:100 proportion: (a) Miglyol® 812; and (b) Isopropyl myristate

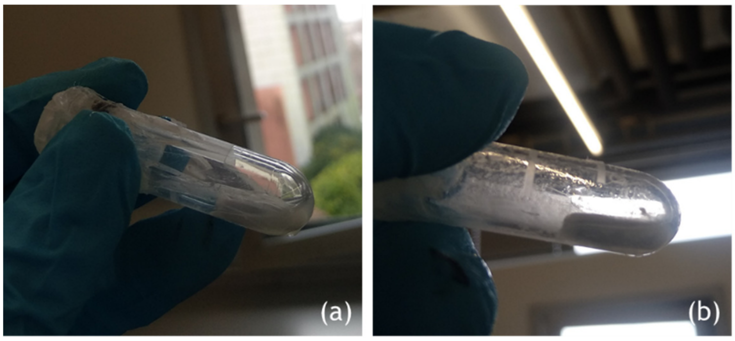

Figure S1. Examples of lipids that were able to solubilize the NC (**a**), while the (**b**) figures are representatives of the ones that could not solubilize the NC, since compound crystals can be visualized.

Compatibility between (a) Apifil® and Miglyol® 812; and (b) Gelucire® 50/13 and Miglyol® 812

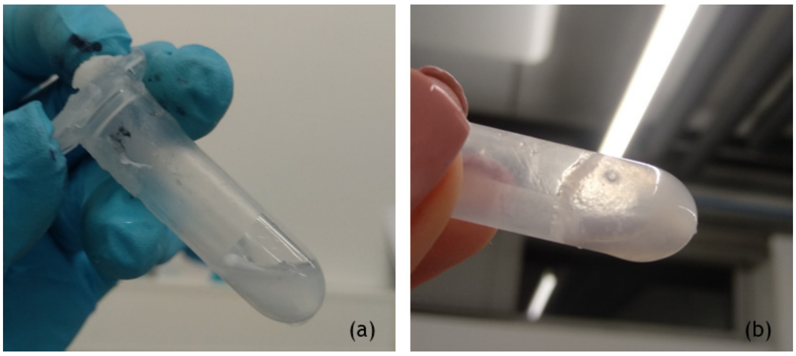

Figure S2. Compatibility evaluation between the solid and liquid lipids. The picture contains an example of the combination where the lipids were compatible (**a**) and where the solubilization did not occur (**b**).

Naringenin-loaded NLCs	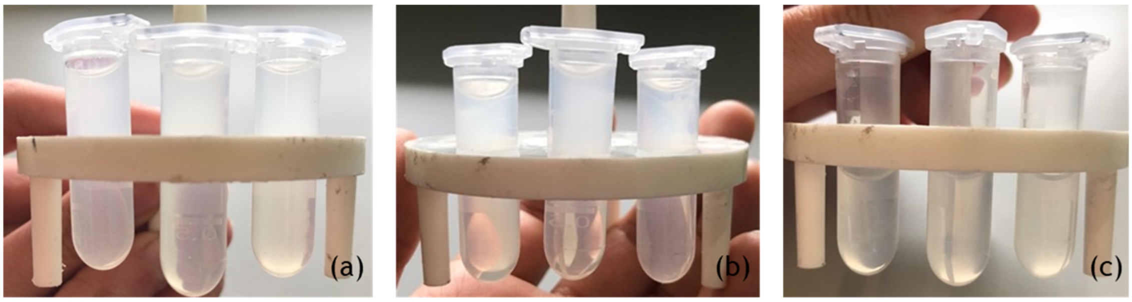
NDGA-loaded NLCs	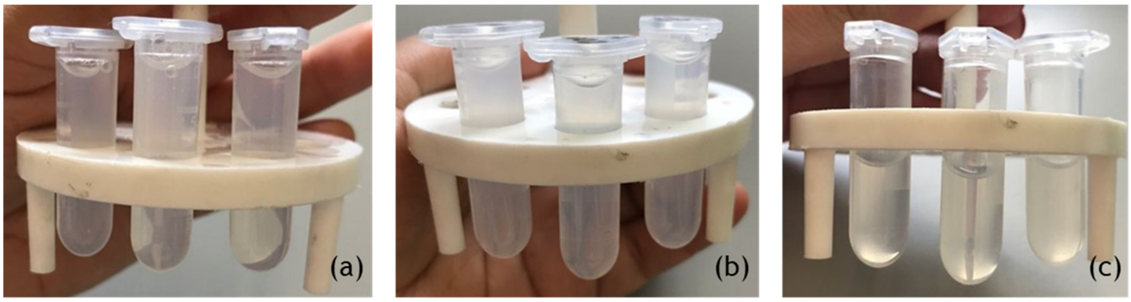
Kaempferol-loaded NLCs	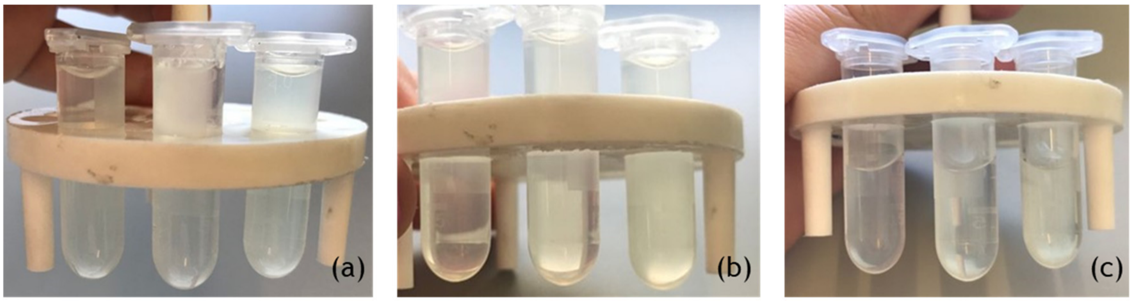

Figure S3. Images of the samples before and after the first and second centrifugation included in the accelerated stability study assays before centrifugation (**a**); after the first centrifugation (30 minutes) (**b**); and after the second centrifugation (30 minutes + 30 minutes) (c).
